# Molecular Epidemiology of Cystic Echinococcosis in Rural Baluchistan, Pakistan: A Cross-Sectional Study

**DOI:** 10.3390/pathogens12010040

**Published:** 2022-12-26

**Authors:** Ihsan Ullah, Sadia Sattar, Ijaz Ali, Arshad Farid, Amin Ullah, Refaat A. Eid, Mohamed Samir A. Zaki, Muhammad Alaa Eldeen, Iftikhar Ahmed, Irfan Ullah

**Affiliations:** 1 Molecular Virology Lab (MVL), Department of Biosciences, COMSATS University Islamabad (CUI), Islamabad 45550, Pakistan; 2 Gomal Center of Biochemistry and Biotechnology, Gomal University, Dera Ismail Khan 29050, Pakistan; 3 Department of Health and Biological Sciences, Abasyn University Peshawar, Peshawar 25000, Pakistan; 4 Pathology Department, College of Medicine, King Khalid University, Abha 62529, Saudi Arabia; 5 Anatomy Department, College of Medicine, King Khalid University, Abha 62529, Saudi Arabia; 6 Department of Histology and Cell Biology, College of Medicine, Zagazig University, Zagazig 31527, Egypt; 7 Biology Department, Cell Biology, Histology & Genetics Division, Faculty of Science, Zagazig University, Zagazig 44519, Egypt; 8 Department of Life Sciences, School of Science, University of Management and Technology (UMT), Lahore 54770, Pakistan

**Keywords:** humans, Pakistan, cystic echinococcosis, molecular epidemiology, *Echinococcus granulosus sensu lato*

## Abstract

Cystic echinococcosis (CE), or hydatid cyst disease (HCD), is a zoonosis of significant importance caused by the cestode of *Echinococcus granulosus sensu lato* (s. l.) that affects mainly nomadic populations and has substantial economic consequences. Due to the 76% rural and nomadic population, Baluchistan is a highly endemic region in Pakistan for CE; however, it has not yet been investigated for CE. For this purpose, this study was carried out to investigate the molecular epidemiology of CE in this region. In total, 23 human hydatid cyst samples were collected from tertiary health care units in Baluchistan and processed for DNA extraction, which was then followed by sequencing of the *cox1* mitochondrial gene of all 23 collected samples, genotyping, and phylogenetic and haplotype analysis. Most subjects were livestock owners (39.13%) in rural settings (73.91%). Most patients (73.19%) were pet owners (dogs) and used water from open sources for drinking. The liver was the most affected organ (52.17%), followed by the lungs (17.39%). Sequence analysis based on the *cox1* gene revealed that EG genotype 1 (G1) was the most prevalent (56.52%), followed by G3 (34.78%), while some samples (8.7%) were identified as the *Echinococcus canadensis* (G6/7) genotype. A total of five haplotypes were detected with high haplotype diversity (0.80) and low nucleotide diversity (0.033). Phylogenetic analysis revealed two diverse sub-clades, each of G1 and G3 isolates from Baluchistan, that were evolutionarily related to previously reported G1 and G3 isolates from Pakistan and China. On the other hand, the G6/7 isolates of this study were evolutionarily identical to the already reported G6/7 isolates from Pakistan, Turkey, and Kazakhstan. This study concludes that diverse G1 and G3 EG isolates are present in this part of Pakistan, while the G6/G7 genotype was reported for the first time from Baluchistan.

## 1. Introduction

Human CE, or hydatid cyst disease, is caused by the larval stage of the tapeworm *Echinococcus granulosus sensu lato* (s. l.), and transmission occurs via the eggs of the definitive host (canids), which are released in the feces and then infect other wild animals and livestock (intermediate hosts) [1]. Humans are the accidental hosts of this parasite and can acquire infection through close interaction with dogs or through ingesting contaminated water and food. EG is a cryptic species that displays significant intraspecific and mitochondrial haplotype heterogeneity [2], and it is now classified mainly based on the host these genotypes infect. It is classified into five different species, four of which (*E. granulosus sensu stricto* (Genotype 1–3), *E. ortleppi* (G5), *E. canadensis* cluster (G6–G10), and *E. equinus* (G4)) are considered infectious to humans and one of which (*E. felidis*) is considered to be non-infectious to humans, and no cases have been reported yet [3,4,5,6].

It is difficult to understand the etiology of echinococcosis as several host species and genotypes/strains are involved in its transmission [7]. The genetic diversity among these strains and species has been attributed to contamination patterns of the definitive host and potential intermediate hosts, along with immunological interactions and responses between host and parasite [8,9]. When many species of echinococcus are present in the same geographical location, the interaction between different transmission cycles can occur as the definitive host is susceptible to several echinococcal species that can infect different intermediate hosts present in that region, which thus can also result in species diversity co-infection [2,3]. Molecular characterization of nuclear and mitochondrial genes has greatly facilitated the process of genotypically characterizing the genus echinococcus [10,11], which has helped to identify and understand this parasite’s transmission patterns [12]. Mitochondrial genes such as *NADH dehydrogenase 1* (*NaDH1)*, *Cyclooxygenase 1* (*cox1)*, *ATP synthase subunit 6* (*Atp6)*, *cytochrome b gene* (*Cob),* and *Large subunit* (*LSU)* are considered ideal markers for the molecular epidemiology of echinococcus. Among these markers, *cox1* is highly recommended for evolutionary and molecular analysis of echinococcus as it has low repetitive sequences, is haploid, and is semi-conserved [2].

Most of the population in Pakistan depends on agriculture and livestock rearing for their livelihoods and daily lives in rural communities, which makes it a higher risk area for CE, as is also the case in neighboring countries such as Iran, China, Afghanistan, and other central Asian states where 270 million people face an increased risk of acquiring this disease [13]. The epidemiological picture of CE is still unclear in humans in Pakistan, although the infection history dates back to the pre-partition era [14]. A limited number of studies have been carried out in the provinces of Sindh, Punjab, and KP, which reported G1 genotype as the most prevalent. At the same time, G3 and G6/G7 genotypes have also been reported [15,16,17,18]. However, there has been no genotypes reported from Baluchistan province, which accounts for almost half of the sheep and 41% of the camel population of Pakistan. In this province, the majority of people rear sheep and other livestock, live in rural settings, have dogs as pets, and use open water sources for drinking. For this purpose, this investigation was carried out to study the molecular epidemiology of CE in humans in Baluchistan, a region of interest in Pakistan that has been neglected.

## 2. Materials and Methods

### 2.1. Area of Study

**Figure 1:**
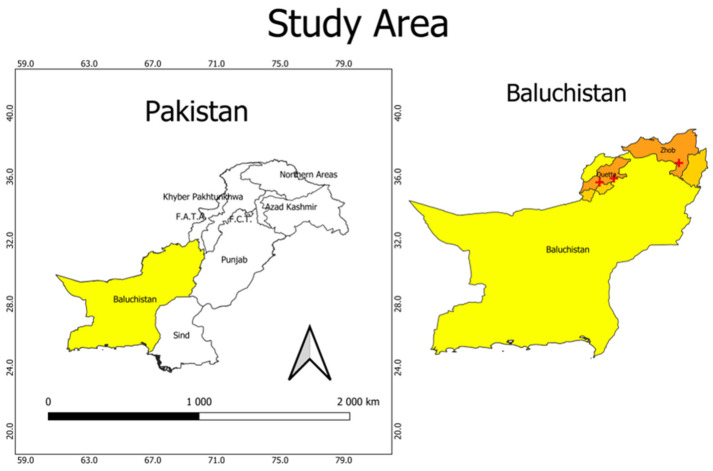
Map of Baluchistan showing the location of the study hospitals (+).

Baluchistan is the largest province of Pakistan area-wise with a total area of 347,190 square kilometers, as shown in Figure 1. At the same time, it is the smallest province population-wise, with a population of 12,335,129, among which 8,928,428 (72.38%) have rural residences and 3,406,701 (27.62%) live in urban settings [19]. Rangelands constitute the major area (approximately 93%) of Baluchistan province, which is crucial for rearing ruminants and camels in this region. Baluchistan shares its border with Sindh, Khyber Pakhtunkhwa, and Punjab to the southeast, northeast, and east, respectively. At the same time, the Arabian Sea lies to the south, Iran to the west, and Afghanistan to the northwest.

### 2.2. Sample Collection

Sample collection was carried out from three main hospitals in Baluchistan, i.e., CMH (Combined Military Hospital) located in Zhob, Civil Hospital, and Bolan Medical Complex Hospital situated in the capital of Baluchistan (Quetta). A total of 23 cyst samples were collected from the surgical units of these hospitals between 2018 and 2020. The cyst samples were kept in 96% ethanol and transported to the Molecular Virology Lab at Comsats University Islamabad for further analysis.

### 2.3. Informed Consent

Written informed consent was obtained from all study participants after briefly discussing the research study and its objectives. Patients under 16 years of age were considered if either of their parents signed the consent form on the child’s behalf.

### 2.4. Histopathology

The collected cyst samples were confirmed as hydatid cysts through histopathological examination due to the presence of protoscoleces, hooklets, and laminated layers. This was followed by classification as either fertile or sterile based on the presence and absence of protoscoleces and hooklets, respectively.

### 2.5. Demographics and Clinical Data

Age, gender, profession, ethnicity, drinking water source, and presence or absence of pets in the house were recorded using the answers from each patient’s questionnaire. Cyst location was also recorded. The hepatic cysts were further classified as CE1, CE2, CE3b, CE3a, CE4, or CE5 according to the WHO-IWGE classification criteria [20].

### 2.6. DNA Isolation

Genomic DNA extraction was successfully performed from germinative layers or protoscoleces from all the collected cyst samples using a commercially available DNeasy Blood and Tissue Kit (Qiagen, Hilden Germany). Extraction was carried out according to the protocol and instructions provided by the manufacturer, and the quality of extracted DNA was evaluated with a NanoDrop 1000 (Thermo Fisher Scientific, Massachusetts USA). The extracted DNA was then stored at low temperature (−20 °C) to avoid degradation before further analysis.

### 2.7. Sequencing

EgCOI1 (TTTTTTGGCCATCCTGAGGTTTAT) and EgCOI2 (TAACGACATAACATAATGAAAATG) were used for PCR amplification of the *cox1* mitochondrial region of the isolated DNA as described by [21] with some modifications. The amplified products were then run on 1.7% agarose gel that yielded a product of 396 base pairs. The PCR product was carefully cut from the gel and purified for sequence analysis using a QIAquick Gel Extraction Kit (Qiagen, Germany). The purified product was then commercially sequenced (Macrogen, Seoul Korea).

The nucleotide sequences obtained in the current study were submitted to GenBank and published under accession numbers ON833443 to ON833465 for samples PKBH1 to PKBH23.

### 2.8. Phylogenetic Analysis

The resulting sequences were then compared to already available sequences using the BLAST tool (basic local alignment search tool) developed by the NCBI (National Centre for Biotechnology Information database) and the accession numbers of highly similar sequences were retrieved, as shown in Table 1 along with the country of origin and source of isolation. Reference sequences were retrieved through Web Query Genbank options in Mega 11 and added to the dataset for phylogenetic analysis.

The ClustalW algorithm in Mega 11 was used for multiple sequence alignment of the sequences obtained in this study and the reference sequences. After manually trimming the extra sequences at both sides, a uniform size of 336 base pairs was obtained for all sequences. These sequences were then used for phylogenetic analysis in Mega 11 [22], and a phylogenetic tree was constructed from a total of 44 sequences by using the neighbor joining method [23] with 1000 bootstrap replicates [24]; evolutionary distances were calculated through the Kimura-2 parameter method [25].

### 2.9. Haplotype Analysis

Haplotype analysis of all 42 sequences in the dataset was carried out using DnaSP 6 software [26]. Divergence and polymorphism between populations, the number of haplotypes, haplotype diversity (Hd), the average number of nucleotide differences (k), nucleotide diversity (Pi), singleton and parsimony informative sites, and Tajima’s D and Fu’s Fs statistics were calculated using DnaSP6. A nexus file was also generated by DnaSP6 for further analysis. PopART-1.7 software http://popart.otago.ac.nz (accessed on 18 July 2022) [27] was used to draw a haplotype network based on the agglomerative hierarchical clustering approach, also known as the bottom-up approach, using the TCS criteria [28].

## 3. Results

### 3.1. Demographics of Patients and Clinical Characteristics of Cysts

A total of 23 human CE samples were collected from the surgical units of tertiary hospitals in Baluchistan from 2018 to 2020. More cases of CE (43.42%) were reported in the year 2020 compared to 2018 (21.74%) and 2019 (34.78%). The majority of the patients were female (56.52%). The highest number of patients (34.78%) belonged to the 31–40 age group. The Baluch ethnic group accounted for most of the cases (60.87%). Most of the patients (39.13%) were livestock bearers, while 73.91% had dogs in their houses, had close interaction with dogs, and also used open water sources for drinking water. A majority of the patients (73.91%) belonged to rural areas. Organically classified cysts showed that several cysts (52.17%) were hepatic (CE3 = 5, CE4 = 5, CE5 = 1), while classification for one cyst was unavailable. Microscopic examination of the cysts showed that 19 (82.61%) were fertile with visible hooks and protoscolices, while the remaining 4 (17.39%) were sterile with no visible hooks or protoscolices. In detail, the demographics of patients, risk factors, and characteristics of the cysts are listed in Table 2.

### 3.2. Genotypic Analysis

Sequence analysis showed that the majority of the human CE samples (13/23 (56.52%)) belonged to the G1 genotype, 10/23 (34.78%) of the samples were detected as the G3 genotype, and the remaining 2/23 (8.7%) were detected as the G6/G7 genotype (*E. canadensis*).

### 3.3. Phylogenetic Analysis

**Figure 2:**
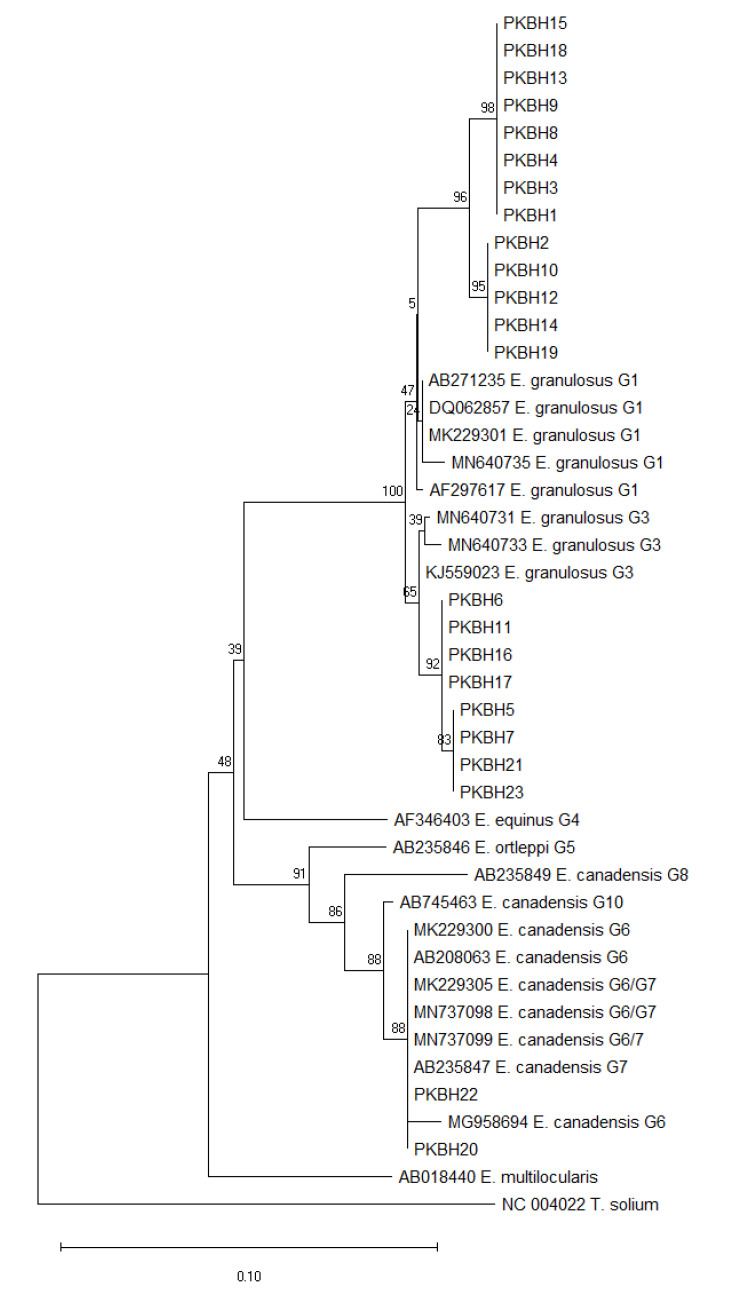
Phylogenetic/evolutionary analysis was inferred using the neighbor joining method. The reliability of the tree was assessed using 1000 bootstrap replicates, and the bootstrap values calculated are shown above the branches. The calculated evolutionary distances are illustrated by the units of the number of base substitutions per site. The isolates of the present study are represented by PKBH1-PKBH23, and the reference sequences are represented by their accession numbers and their genotypes. The scale bar is representative of the number of substitutions per site.

Phylogenetic analysis revealed three distinct clades (G1, G3, and G6/7) of EG isolates in this study, as shown in Figure 2. The G1 clade was divided into two sub-clades with a high bootstrap value of 96 at the node. The G1 EG isolates of this study were evolutionarily related to human EG isolates reported from other regions of Pakistan and China, as well as sheep and dog EG isolates reported in Pakistan, Italy, and Mongolia. The G3 clade was divided into two sub-clades with a significant bootstrap value of 92. The EG G3 isolates reported in this study were evolutionarily related to other EG G3 isolates reported from sheep and buffalo in Pakistan, as well as human EG isolates from China. The G6/7clade also has a high bootstrap value of 88. The human *E. canadensis* (G6/7) isolates from this study are evolutionarily identical to human, camel, dog, and pig G6/7 isolates from Pakistan, Kazakhstan, Turkey, and Japan, i.e., MK229300, AB208063, MK229305, MN737098, MN737099, and AB235847, as shown in Figure 2.

### 3.4. Haplotypes

A total of 16 haplotypes were detected in this study, and haplotype diversity was recorded as 0.91. Out of these 16 haplotypes, 5 haplotypes were detected in the human EG isolates of this study, with a haplotype diversity of 0.80 (Supplementary Table S1). Hap_4 and Hap_5 in the present study belonged to the G1 genotype consisting of eight (PKBH1, PKBH3, PKBH4, PKBH8, PKBH9, PKBH13, PKBH15, and PKBH18) and five EG isolates (PKBH2, PKBH10, PKBH12, PKBH14, and PKBH19), respectively. Hap_4 and Hap_5 had four polymorphic sites at positions 84, 179, 265, and 296. The other two haplotypes detected in this study, Hap_9 and Hap_10, belonged to the G3 genotype consisting of four human EG isolates and had a single polymorphic site at position 80. Hap_12 belonged to the G6/7 genotype and consisted of two EG isolates from this study and six reference G6/7 isolates that were identical. The haplotypes detected in the study are shown in Table 3.

Polymorphism of the studied isolates showed low nucleotide diversity (Pi) of 0.03 for the *cox1* gene and an average number of nucleotide differences (K) of 11.97, while nucleotide diversity was 0.055 for the dataset with a K value of 18.55, as shown in Table 4. Tajima’s D score was negative (−0.171), and Fu’s Fs statistics was positive (1.34) but non-significant for isolates of this study. Tajima’s D was positive (0.131) and Fu’s Fs was negative (−0.75) and non-significant for the dataset including isolates of this study along with reference isolates (Table 4).

**Figure 3:**
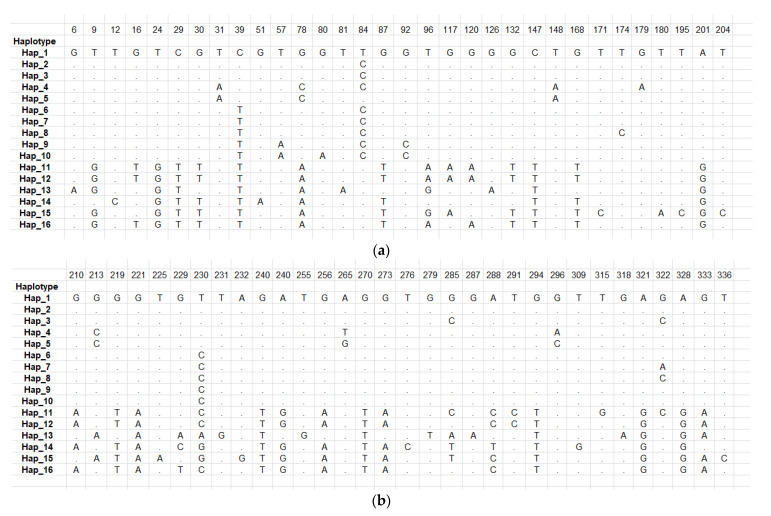
(**a**,**b**) Polymorphic (variable) sites among different haplotypes.

A total of 77 mutations and 64 polymorphic sites were detected in our dataset; 23 isolates were from this study and 19 were reference isolates, out of which 22 were singleton variable sites. Additionally, 42 were parsimony informative sites, as shown in Table 5. Among the 23 EG isolates of this study, 40 polymorphic sites and 43 mutations were detected that were parsimony informative sites, while there were no singleton variable sites in these sequences. Polymorphic sites, along with their site positions, are illustrated in Figure 3a,b.

### 3.5. Haplotype Network

**Figure 4:**
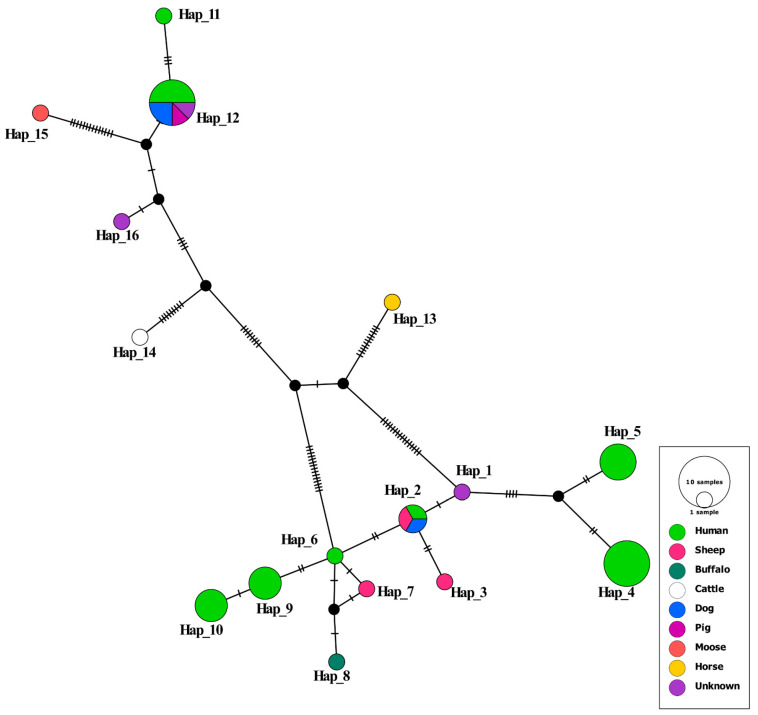
Haplotype network analysis of the samples and references.

The 16 haplotypes detected in this study formed a star-shaped network with 3 central haplotypes, i.e., hap_1 for the G1 genotype that belonged to human EG isolates from China, Hap_6 for the G3 genotype that also belonged to human EG isolates from China, and hap_12 for the G6/G7 genotype that consisted of a total of 8 EG isolates—of which 4 were EG isolates from humans (2 from this study and 2 from previous studies), 2 were EG dog isolates from Turkey, 1 was an EG camel isolate from Kazakhstan, and 1 was an EG pig isolate from Japan. The two haplotypes detected in this study as G1 isolates of EG (Hap_4 and Hap_5) were very far away from the central haplotype Hap_2. Similarly, Hap_9 and Hap_10 were detected as G3 isolates of EG, which also occurred at a significant distance from the central haplotype Hap_6. In contrast, the two samples detected in this study as G6/7 isolates of EG exist within the central haplotype for G6/G7 (Hap _12) as shown in Figure 4.

## 4. Discussion

The first scientific reports on the existence of CE in Pakistan date back to 1938 [14]; however, very few research efforts have been undertaken to understand its epidemiology, especially among the affected human population. A couple of studies have recently reported CE in human populations from different regions of Pakistan, including the provinces of Punjab [17,29], Sindh [18], and Khyber Pakhtunkhwa [15]; however, the molecular epidemiology of CE in the human population in the province of Baluchistan has never been investigated.

This study was carried out to elucidate the current molecular epidemiological status of CE in the human population of Baluchistan. The incidence of CE was highest in 2020, followed by 2019 and 2018, which signifies that the number of CE cases is increasing in humans in this region.

The G1 genotype of CE was previously reported as the most prevalent genotype among humans in Pakistan [18,29]. At the same time, it has also been reported in the majority of human cases in China [30] and Iran [31], which border Pakistan. The G1 genotype has also been reported as the major causative agent of human CE worldwide [32]. Sheep comprise more than half of the livestock population in Baluchistan and the majority of herd keepers also keep dogs with their herds, while stray dogs are also abundant in the region, which makes people more susceptible to infection by CE due to the sheep–dog life cycle of the G1 genotype of EG [33]. G3 was the second most prevalent genotype of EG in Baluchistan in this study. Other studies have also reported G3 to be the second most prevalent genotype of EG in humans [18]. Buffaloes are considered the ideal host for the G3 genotype of EG [34]. The buffalo population was previously low in Baluchistan but the trend is now shifting with the establishment of dairy farms in peri-urban areas, for which stock is mainly brought from Punjab where G3 has also been reported to be the most prevalent (78.75%) genotype of EG in cattle [17]; this might be the reason for detection of the G3 genotype in this region.

The G6/7 genotype of EG, which was first considered non-infectious to humans, was also reported in this region. However, cases are now being reported regularly from different areas of Pakistan, including the Punjab [29], Khyber Pakhtunkhwa [15], and Sindh [18] provinces of Pakistan, which suggests that this genotype is now circulating among the human population and becoming more infectious with time. Baluchistan has 41% of the total camel population of Pakistan, which itself has the eighth-largest population of camels worldwide [35]. A previous study has reported the prevalence of hydatidosis in camels to be 35.92% [36]. The high population of dogs and reliance on open water drinking sources can lead to increased infection from the G6/7 genotype of EG in humans; however, few cases have been reported in the current study and the role of other intermediate hosts in spreading this genotype is still unclear, though camels are most likely the source of the cause. Additionally, Baluchistan shares a long open border with Iran, and frequent illegal trade, including the trade of livestock, occurs throughout the year, which may also have contributed to the detection of this genotype in the region as infection with the genotype has been frequently reported in Iran [15,16]. This also increases the risk of co-infection and further genetic variations among the different genotypes of EG found in the region.

Incidence of CE in females was reported to be high in other studies [15,37,38], which is in line with our results and can be attributed to their close interaction with livestock and domestic dogs (pets) as, in the majority of rural households, females are responsible for taking care of livestock in this region. People from the 31–40 age group, especially females, are usually caretakers of their families and manage households, which includes the rearing of livestock. This is why this age group might be the most infected among the study population; the same age group (31–40) was also reported to be the most affected in Baluchistan by another study, with 22.7% of cases [38]. Unlike our results, other studies from Pakistan have reported the 21–30 age group as the most infected group [15,18]. CE has long been reported to be a zoonosis of rural areas [6], and another study also reported higher prevalence (76.3%) of CE in rural regions of Pakistan [29], which is consistent with our results. Similarly, most patients also had dogs as pets in their houses, had close interaction with dogs, and used water from open sources for drinking, which is considered to be a risk factor for CE [8,39] as the drinking water may be contaminated with EG eggs. In another study Khan et al. [15], reported a higher prevalence of CE in humans from Pakistan who had close interaction with dogs.

In our study, the majority of cases were found in the liver, which has already been reported as the main organ infected by CE [18,29]. The WHO-IWGE classification of hepatic cysts plays a critical role in the management of CE as it corresponds to cyst activity (active, inactive, or transitional); however, it is neglected in Pakistan and other developing countries due to a lack of expertise. Even stage CE4 and CE5 cysts are removed via surgery, which is not in line with WHO-IWGE guidelines as surgery for cysts of these stages is not recommended, with a focus on treatment and an observe–wait approach recommended instead. There is a dire need to develop and implement a national strategy for surgeons based on the WHO-IWGE classification [20] as imaging techniques have advanced significantly, and this strategy would thus reduce disease burden by reducing the number of surgeries for CE patients.

Phylogenetic analysis revealed that the two EG isolates detected as belonging to the G6/7 genotype were 100% identical with previously identified G6/7 isolates in humans in other regions of Pakistan (MK229300 and MK229305) [29]. In addition, they were similar to the G6/7 isolates identified in camel, dog, and pig isolates in Kazakhstan, Turkey, and Japan [40,41], which suggests that a common G6/7 genotype of EG may be circulating in humans, dogs, and other intermediate hosts in Pakistan and other countries in Asia. Evolutionary analysis revealed that G1 and G3 isolates in this study were related to, but somewhat diverse from, already reported isolates in humans, buffalo, and sheep from other regions of Pakistan [18,29], as well as those reported in China, Italy, and Mongolia [42,43,44]. This divergence may be attributed to either a founder event or vicariance, as the EG lineages may have evolved separately and become further distinct with time. The current analysis revealed high haplotype diversity and low nucleotide diversity (signifying high genetic variability and the high value of a population’s genetic structure), which can be attributed to the presence of two G6/G7 genotypes in the samples. The negative Tajima’s D score for the EG isolates of this study signifies an expansion of the population due to possible purifying selection. The positive Fu’s Fs value for EG isolates signifies a deficiency of alleles due to a recent population bottleneck.

CE significantly impacts the lives and economies of the human population; however, very little research has been directed toward understanding its epidemiology and management in endemic areas, especially in Pakistan and Baluchistan. Further research is needed to investigate the prevalence of CE in different domestic animals, wild animals, and humans in this region, which could help deepen the understanding of CE’s etiology and management. Furthermore, the lack of public awareness increases the risk of transmission not only to humans, but also to livestock. These practices also increase the risk of mutations and the transmission of new genotypes within human and livestock populations.

## 5. Conclusions

The G1 genotype of EG was reported to be the most prevalent genotype followed by the G3 genotype, while the G6/G7 (*E. canadensis*) genotype was also reported for the first time in this region. The reported EG isolates in this study had high similarity to Chinese EG isolates. Risk factors such as pet and livestock ownership, rural settings, and a reliance on drinking water from open sources were identified as compounding factors for the prevalence of diverse EG strains. Further studies and preventive measures are required at provincial and national levels to investigate genotypes prevalent in other intermediate hosts and mitigate the spread of CE in high-risk groups in this region.

## Figures and Tables

**Table 1:** Reference sequences used in the current study, along with the source and country of isolation.

References	Genotype	Isolation Source	Country
AF297617	G1	Human	China
AB271235	G1	Dog	Mongolia
DQ062857	G1	Sheep	Italy
MK229301	G1	Human	Pakistan
MN640735	G1	Sheep	Pakistan
KJ559023	G3	Human	China
MN640731	G3	Sheep	Pakistan
MN640733	G3	Buffalo	Pakistan
MG958694, MK229300	G6	Human	Pakistan
AB208063	G6	Camel	Kazakhstan
MK229305	G6/G7	Human	Pakistan
MN737098, MN737099	G6/G7	Dog	Turkey
AB235847	G6/G7	Pig	Japan
AF346403	G4	Horse	Australia
AB235846	G5	Cattle	Japan
AB235849	G8	Sheep	China
AB745463	G10	Deer	Finland

**Table 2:** Patient demographics and the characteristics of cysts.

Demographics of Patients			
Characteristics	Category	n (%)	
**Gender**	Male	10 (43.48)	
	Female	13 (56.52)	
**Ethnicity**	Baluch	14 (60.87)	
	Pashtun	9 (39.13)	
**Profession**	Livestock rearing	9 (39.13)	
	Butcher/livestock	2 (8.7)	
	Farming/livestock	2 (8.7)	
	Student/livestock	3 (13.04)	
	Other	7 (30.43)	
**Pet**	Yes	17 (73.91)	
	No	6 (26.09)	
			**Gender**
**Age Groups**	0–10	2 (8.7)	M = 1, F = 1
	11–20	3 (13.04)	M = 1, F = 2
	21–30	3 (13.04)	M = 1, F = 2
	31–40	8 (34.78)	M = 4. F = 4
	41–50	4 (17.39)	M = 1. F = 3
	51–60	2 (8.7)	M = 1, F = 1
	≥61	1 (4.35)	M = 1. F = 0
**Residence**	Urban	6 (26.09)	
	Rural	17 (73.91)	
**Drinking Water Source**	Open water/shared	17 (73.91)	
	Tap water	6 (26.09)	
**Cyst Characteristics**			
**Fertility of Cyst**	Fertile	19 (82.61)	
	Sterile	4 (17.39)	
			**WHO classification of hepatic cysts**
**Cyst Location**	Liver	12 (52.17)	CE3 = 5, CE4 = 5, CE5 = 1, Not available = 1
	Lungs	4 (17.39)	
	Gall bladder	2 (8.7)	
	Spleen	2 (8.7)	
	Abdomen	2 (8.7)	
	Breast	1 (4.35)	

**Table 3:** Haplotypes detected in the present study with Hd.

Haplotype	Samples	No. of the Samples (n)	Genotype	Haplotype Diversity
Hap_1	AF297617	1	G1	0.91
Hap_2	AB271235, DQ062857, MK229301	3	G1	
Hap_3	MN640735	1	G1	
Hap_4	PKBH1, PKBH3, PKBH4, PKBH8, PKBH9, PKBH13, PKBH15, PKBH18	8	G1	
Hap_5	PKBH2, PKBH10, PKBH12, PKBH14, PKBH19	5	G1	
Hap_6	KJ559023	1	G3	
Hap_7	MN640731	1	G3	
Hap_8	MN640733	1	G3	
Hap_9	PKBH6, PKBH11, PKBH16, PKBH17	4	G3	
Hap_10	PKBH5, PKBH7, PKBH21, PKBH23	4	G3	
Hap_11	MG958694	1	G6	
Hap_12	MK229300, AB208063, MK229305, MN737098, MN737099, AB235847, PKBH22, PKBH20	8	G6/G7	
Hap_13	AF346403	1	G4	
Hap_14	AB235846	1	G5	
Hap_15	AB235849	1	G8	
Hap_16	AB745463	1	G10	

**Table 4:** Diversity and neutrality indices of the studied samples and references.

				Diversity Indices				Neutrality Indices	
Population	N	Eta (No. of Mutations)	K	Pi	Hd	Theta/Seq from Eta	Theta/Site from ETA	Tajima’s D	Fu’s Fs
Samples	23	43	11.14	0.033	0.80	11.65	0.035	−0.171	1.34
Samples with references	42	77	18.55	0.055	0.91	17.89	0.053	0.131	−0.75

**Table 5:** Polymorphic sites and the number of mutations.

Population	N	Number of Mutations (Eta)	Polymorphic Sites	Singleton Variable Sites	Parsimony Informative Sites
Samples only	23	43	40	0	40
All samples with all references	42	77	64	22	42

## Data Availability

The demographics of patients and other relevant data that was collected, generated, and analyzed in this study are available in the paper, while some information like name, address, and other contact information of the patients were anonymized. The nucleotide sequences the Cox-1 gene of all 23 samples have been submitted and published under the accession number ON833443 to ON833465, respectively, in GenBank.

## Supplementary Materials

The supplementary data can be downloaded at https://www.mdpi.com/article/10.3390/pathogens12010040/s1, Table S1: Haplotypes detected in the study samples.
