# Supplementary material for: Molecular Epidemiology of Cystic Echinococcosis in Rural Baluchistan, Pakistan: A Cross-Sectional Study
Source: Pathogens. 2022 Dec 26;12(1):40. doi: 10.3390/pathogens12010040 (PMC9863174; doi:10.3390/pathogens12010040)
Supplement: Supplementary file 1 [file pathogens-12-00040-s001.zip › pathogens-2013258-supplementary.pdf]

Table S1. Haplotypes detected in the study samples.

Haplotype	Samples	No of Samples (n)	Genotype	Haplotype diversity
Hap_1	PKBH1. PKBH3. PKBH4. PKBH8. PKBH9. PKBH13. PKBH15. PKBH18	8	G1	
Hap_2	PKBH2, PKBH10, PKBH12. PKBH14, PKBH19	5	G1	0.80
Hap_3	PKBH6, PKBH11, PKBH16, PKBH17	4	G3	
Hap_4	PKBH5, PKBH7, PKBH21, PKBH23	4	G3	
Hap_5	PKBH22, PKBH20	2	G6/G7	
